# Schistoscope: An Automated Microscope with Artificial Intelligence for Detection of *Schistosoma haematobium* Eggs in Resource-Limited Settings

**DOI:** 10.3390/mi13050643

**Published:** 2022-04-19

**Authors:** Prosper Oyibo, Satyajith Jujjavarapu, Brice Meulah, Tope Agbana, Ingeborg Braakman, Angela van Diepen, Michel Bengtson, Lisette van Lieshout, Wellington Oyibo, Gleb Vdovine, Jan-Carel Diehl

**Affiliations:** 1 Delft Center for Systems and Control, Faculty of Mechanical, Maritime and Materials Engineering, Delft University of Technology, 2628 CD Delft, The Netherlands; p.o.oyibo@tudelft.nl (P.O.); t.e.agbana@tudelft.nl (T.A.); g.v.vdovine@tudelft.nl (G.V.); 2 ANDI Centre of Excellence for Malaria Diagnosis, College of Medicine, University of Lagos, Lagos 101017, Nigeria; woyibo@unilag.edu.ng; 3 Department of Sustainable Design Engineering, Faculty of Industrial Design Engineering, Delft University of Technology, 2628 CE Delft, The Netherlands; saatyyya@gmail.com (S.J.); ingeborgbraakman@gmail.com (I.B.); 4 Department of Parasitology, Leiden University Medical Center, 2333 ZA Leiden, The Netherlands; b.meulah_tcheubousou@lumc.nl (B.M.); a.van_diepen@lumc.nl (A.v.D.); m.l.bengtson@lumc.nl (M.B.); e.a.van_lieshout@lumc.nl (L.v.L.); 5 Centre de Recherches Medicales des Lambaréné, CERMEL, Lambarene BP 242, Gabon

**Keywords:** diagnosis, digital microscope, slide scanner, autofocus, artificial intelligence, distributed manufacturing, low resources settings, *Schistosoma*, parasites

## Abstract

For many parasitic diseases, the microscopic examination of clinical samples such as urine and stool still serves as the diagnostic reference standard, primarily because microscopes are accessible and cost-effective. However, conventional microscopy is laborious, requires highly skilled personnel, and is highly subjective. Requirements for skilled operators, coupled with the cost and maintenance needs of the microscopes, which is hardly done in endemic countries, presents grossly limited access to the diagnosis of parasitic diseases in resource-limited settings. The urgent requirement for the management of tropical diseases such as schistosomiasis, which is now focused on elimination, has underscored the critical need for the creation of access to easy-to-use diagnosis for case detection, community mapping, and surveillance. In this paper, we present a low-cost automated digital microscope—the Schistoscope—which is capable of automatic focusing and scanning regions of interest in prepared microscope slides, and automatic detection of *Schistosoma haematobium* eggs in captured images. The device was developed using widely accessible distributed manufacturing methods and off-the-shelf components to enable local manufacturability and ease of maintenance. For proof of principle, we created a *Schistosoma haematobium* egg dataset of over 5000 images captured from spiked and clinical urine samples from field settings and demonstrated the automatic detection of *Schistosoma haematobium* eggs using a trained deep neural network model. The experiments and results presented in this paper collectively illustrate the robustness, stability, and optical performance of the device, making it suitable for use in the monitoring and evaluation of schistosomiasis control programs in endemic settings.

## 1. Introduction

Bright-field microscopy is still the dominant method for imaging in numerous engineering and scientific domains as a result of its accessibility. Of particular interest to this work is the medical discipline of diagnostics, in which it is still the reference standard procedure for diagnosis and load estimation for many infectious diseases, particularly those caused by parasites [1]. 

Schistosomiasis is a neglected tropical disease (NTD) [2] caused by the parasitic flatworm called *Schistosoma*. Approximately 700 million people living in ~80 countries are at risk of infection, of which around 90% live in Africa [2,3,4]. Several *Schistosoma (S)* species can infect humans, with *S. haematobium* being one of the most prevalent species in Africa and the cause of urogenital schistosomiasis [5]. The reference standard procedure for the diagnosis of *S. haematobium* infection is the detection of eggs in urine via microscopic examination, while counting the number of eggs in a specified volume of urine (quantitative analysis) is used for epidemiological surveillance [3,4,5,6,7]. One of the major limitations of this procedure is that it is operator-dependent, meaning it is prone to discrepancies in performance since expertise and skills can vary across individuals [8,9]. Furthermore, since infections are predominantly found in rural settings in poor-resource regions, the availability of functioning microscopes can be a challenge [10]. Additionally, the employment of skilled microscope operators is costly and requires investment in ongoing training [8,9]. Furthermore, the diagnostic performance of this procedure is inversely related to the number of worms present, meaning that light infections with low egg excretion will be easily missed, while these individuals can still contribute to the transmission of the disease [10]. Finally, the current World Health Organization’s (WHO) agenda to eliminate neglected tropical diseases (including schistosomiasis) [11], requires the precision mapping of communities and conventional microscopy, which is mostly used in resource-constrained settings where schistosomiasis is endemic, may not be able to accelerate this elimination agenda. The critical need for periodic monitoring of interventions in communities at the ward level will require devices with automation and self-diagnostic capacities that human operators alone may not be able to readily provide.

For these reasons, there is a need for inexpensive and smart portable devices capable of slide-scanning and performing digital microscopic examination. Such a device will ensure better and consistent performance across diagnosis, speed up sample scanning, compensate for the lack of trained microscope operators in some countries, and assist in diagnosis using artificial intelligence algorithms where needed. When used with an onboard computer, regional epidemiological data can potentially be uploaded to a database, therefore allowing stakeholders involved in epidemiological surveillance to plan and authorize control and elimination schemes. Moreover, such devices will ease the workload on microscopists in epidemiological surveys or impact assessment programs, where there are a lot of samples to be analyzed, thus minimizing errors in diagnosis.

In recent years, accessibility of manufacturing methods such as 3D printing and laser-cutting has increased. Furthermore, the availability of smartphones, which have an in-built camera, and miniature computers such as Raspberry Pi have also increased. This growth has led to the development of computerized instruments. 

Studies on smartphone-based microscopes have been reported [12,13,14,15,16] with optical setup with numerical aperture (NA) and magnification equivalent to or higher than some microscope objectives. However, these microscopes do not have mechanical stages, making it challenging to maintain focus while manually changing the field of view (FoV). On the other hand, the scientific literature also includes studies that has developed open-source microscope designs, automated movement of the XYZ sample stages and microscope objectives [17,18,19,20]. One notable design is the open-source OpenFlexure device by Collins et al., which uses a 100× microscope objective and was applied to clearly resolve malaria parasites in thin blood smears [21]. Li et al. developed a highly configurable instrument at a variable cost of USD 250–500 (depending on the configuration) that is capable of quantifying malaria parasites by scanning 1.5 million red blood cells per minute [22]. There have also been studies on automatic *S. haematobium* egg detection which focus mainly on identifying eggs in images pre-captured by professional clinical operators mostly with isolated and non-overlapping eggs in an FoV [23,24,25]. Essentially, captured images of urine samples prepared in field settings often contain a lot of artifacts such as crystals, glass debris, air bubbles, fabric fibers and human hair. Thus, an automatic *S. haematobium* egg detection system applicable in field settings remains unexplored.

In this work we demonstrate the potential for a low-cost yet high-quality instrument, called the Schistoscope, that can function as a reliable digital microscope, slide scanner and an automatic diagnostic tool for use in point-of-need diagnostics. We build on our earlier efforts [26,27,28] with a focus on the detection of *S. haematobium* eggs in urine. The Schistoscope performs autofocusing, automated filter membrane scanning (creating an image grid of the sample) and automatic *S. haematobium* egg detection and count estimation. The four main drivers in the design of the Schistoscope are focused on: (i) the robustness of the device in its ability to withstand the harsh tropical working environment in sub-Saharan Africa, such as humidity, dust and heat; (ii) potential for local production mainly using standard off-the-shelf components in combination with locally available distributed production methods to bring the cost of the device to approximately USD 700 and enable local maintenance and repair; (iii) operational considerations such as the intuitiveness and ease of use; (iv) hygiene considerations in the handling of the device to ensure that the product could be easily cleaned to prevent possible cross-contamination between samples. 

With these factors in mind, our design of the Schistoscope has undergone five design iterations [27,28] with implementation research conducted in the field, involving key stakeholders in the research and development process, where the device will potentially be used. For further proof of principle, we also demonstrate the detection of *S. mansoni* and hookworm eggs in fecal samples prepared using Kato–Katz technique.

## 2. Materials and Methods

### 2.1. Optical System

**Figure 1:**
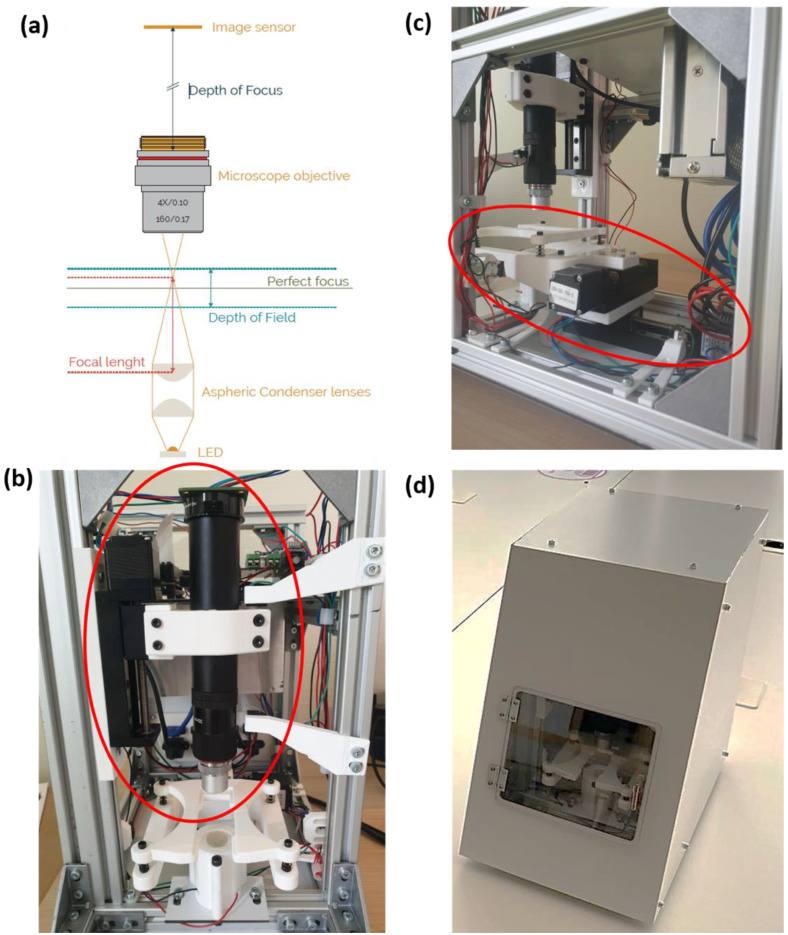
(**a**) Schematic diagram of the Schistoscope optical train (**b**) Region of interest showing the Z-axis consisting of a mechanical slider and optical setup (**c**) Region of interest showing the sample stage mounted on the X and Y slider mechanism (**d**) Exterior of the Schistoscope device (embodiment).

We designed the Schistoscope optical system using the working principle of a conventional light microscope (Figure 1a,b). The illumination system is positioned below the sample stage. Light rays that have passed through the sample are transmitted through the microscope objective lens, which sits just above the specimen, and the image is recorded on the image sensor which is further away from the sample. We employed two convex lenses in the illumination system: the collector lens and the condenser lens. It is designed to provide bright and even illumination on the sample plane and the image plane where the image produced from the objective is recorded by the sensor. This is important because it eliminates glare in the captured image since backlight illumination floods the object with light from behind.

The Schistoscope optical train is similar to that of a standard microscope, except in our design the eyepiece is replaced by a camera sensor, focus adjustment knobs are replaced by an automated Z-axis movement system and software-based autofocus, while the mechanical stage is replaced by automated XY-axis movement systems. With the open-source philosophy in mind, we use an easily accessible and community supported camera module for the Schistoscope—Raspberry Pi High-Quality Camera Module V2.1, equipped with a Sony IMX477R stacked, back-illuminated sensor, 12.3 megapixel resolution, 7.9 mm sensor diagonal and 1.55 μm × 1.55 μm sensor pixel size. We aligned the camera module with a basic achromatic microscope objective using the Thorlabs Extra-Long 6 inches (152.4 mm) extension tube. To visualize *Schistosoma* eggs, we used a 4× magnification objective (with 0.10 numerical aperture, corresponding to a focal length of ~40 mm); however, the device is designed such that the objective is easily interchangeable with a microscope objective up to 20× magnification. Higher magnifications cannot be used due to limited resolution of the Z-axis slider in our design. The illumination system consists of high-power white LED chips welded on a printed circuit board (PCB) and a 25 mm diameter, 20.1 mm focal length Thorlabs aspheric condenser with diffuser. The tube lens is connected to a motorized slider mechanism for effective movement of the optical train along the Z-axis to obtain accurate focus on the sample. In the design of the sample stage, it is important to ensure consistency when moving from one FoV to another in the sample plane to prevent errors during the automatic slide-scanning procedure. Hence, we designed the sample stage as a simplified cantilever beam system, in which the sample holder is mounted on top of an XY stage consisting of two motorized slider mechanisms (similar to the one on the Z-axis) with their individual stepper motors. The Y-axis slider mechanism is fixed, and it translates the X-axis slider mechanism on which the sample holder is directly mounted as shown in Figure 1c.

### 2.2. Electronics System

The Schistoscope makes use of the Raspberry Pi 4 computer board, which provides a high-bandwidth interface to connect the Raspberry Pi camera module. The Raspberry Pi board is also connected to an Arduino Nano board with sufficient general-purpose input–output (GPIO) pins to communicate with other electronic components such as six limit switches positioned at both ends of the X-, Y- and Z-axis, 3 NEMA 11 stepper motors along with their respective controllers for movement along each axis. A custom-made PCB that acts as a shield connects the Arduino board to the various components. We adopted a 60W AC–DC double output switching power supply to power the onboard computer and various device’s electrical components.

### 2.3. Supporting Structures and Enclosure

We adopted aluminum profiles for designing the supporting system to ensure robustness and stability of the device [29]. This will prevent the need for frequent optical system re-calibration and highly trained personnel for system maintenance which is generally unavailable in low-resource settings. Aluminum profiles are widely used for 3D printers, CNC machinery, and research test set-ups. These profiles allow for easy attachment of other systems, and the corner joint allows for quick adaptations in design. The frame is constructed by attaching the profiles with metal corner joints, thus creating rectangle constructions. The frame uses several multiple profiles to allow for change and attachment of an enclosure. The setup creates multiple rectangles to increase rigidness. The bottom profiles prevent the device from tilting forwards, the upper profiles prevent the vertical profiles from leaning, and the middle profiles allow for the mounting of an electronics panel. To prevent the internal system from adverse exposure to external factors such as dust, dampness, or accidental interference by humans, we designed an enclosure system using a material called ‘Alubond’, a lightweight, maintenance-free material. A very low expansion coefficient makes it suitable for temperatures in sub-Saharan Africa. The material allows for production with laser-cutting, CNC-milling, sawing and drilling. The enclosure system is robust and attaches easily to the supporting system. The surface is easy to clean, and the white color resembles a medical device.

### 2.4. Sample Preparation

For this study, *S. haematobium* eggs were obtained from gut tissue of hamsters infected with *S. haematobium* at the Leiden University Medical Center (LUMC) following a standardized protocol approved by the Dutch Central Authority for Scientific Procedures on Animal (CCD) as described previously [30]. Briefly, five weeks after infection with *S. haematobium*, hamsters were sacrificed, and eggs were obtained following gut tissue digestion with collagenase B and extensive tissue washing. Eggs were concentrated in normal saline (5000 eggs per mL) to prevent hatching and stored appropriately for future use. The gut tissue derived eggs are morphologically identical to that seen in human-infected samples. Ten milliliters of urine samples provided by voluntary donors after oral consent were spiked with 0.1, 0.2, 0.3, 0.4, 0.5, 0.6, 0.7, 0.8, 0.9, 1 mL of the concentrated stock (5000 eggs per mL) to make 10 dilutions. 

In addition to the artificially spiked samples, clinical urine and stool samples were obtained during a field study in Federal Capital Territory (FCT), Abuja, Nigeria, in collaboration with the University of Lagos, Nigeria. Ethical approval for this study was obtained from the Federal Capital Territory Health Research Ethics Committee (FCT-HREC) Nigeria (reference No., FHREC/2019/01/73/18-07-19). After receiving informed consent, a total of 33 urine samples were collected in 20 mL sterile universal containers from school-age children who had observed the presence of blood in their urine or had been to the infected community river in the past six weeks. This screening increased the chances of having positive samples for our dataset. The spiked and clinical urine samples were processed using the standard urine filtration procedure [31]. With a syringe, 10 mL of urine was passed through a 13 mm diameter filter membrane with a pore size of 0.2 μm. After filtration, the membrane was placed on a microscopy glass slide, and covered with a coverslip to increase the flatness of the membrane for image capture using the Schistoscope. 

The fecal samples were processed using the standard Kato–Katz procedure with a 41.7 milligram template [32]. The prepared microscopic slides were imaged using the Schistoscope device.

### 2.5. Autofocus and Auto-Scanning System

Microscopic imaging of filter membranes for the detection of *S. haematobium* eggs usually encounters challenges such as uneven filter membranes, presence of artifacts, and deviations of slide angle and stage position. All these factors can result in loss of focus when capturing images across different FoVs, thus reducing the readability of the image by both humans and automatic object detection algorithms. Therefore, there is a need for an autofocusing system to ensure that the images captured are always in focus. We designed the autofocusing algorithm using the following steps [21,22]: first, the microscope objective is moved sequentially through a set of positions along the Z-axis, and at each position an image is captured and converted to greyscale. Next, a sharpness metric is calculated from the edge image derived by applying a 2D Laplacian filter to the grayscale image. The image with the maximum sharpness metric is selected as the image with the best focus. Due to the high resolution of the system, only a limited FoV (1078 µm × 1470 µm) can be imaged at one time point. Therefore, a 13 × 9 grid of images is required to image an entire 13 mm filter membrane for accurate diagnosis. 

**Figure 2:**
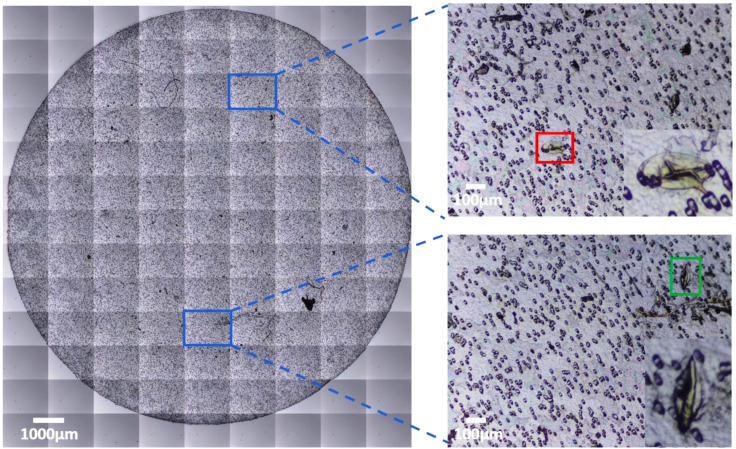
Automated image grid acquisition of *Schistosoma* eggs from a urine filter membrane. Blue region of interest shows individual sub-images, red and green regions of interest are *S. mansoni* and *S. haematobium* eggs, respectively, present in the urine sample. Enlarged areas show the eggs at 300% digital zoom.

We reduced the risk of focusing on the slides by defining the top curvature of the membrane as a starting position for the auto-scanning procedure. The Schistoscope performs auto-scanning in a row-wise traversal order beginning from the upper-left position of the grid using the X and Y slider mechanism. An example grid of a filter membrane captured using the Schistoscope is shown in Figure 2.

### 2.6. Automatic S. haematobium Egg Detection

To automatically detect *S. haematobium* eggs, we first created a large-scale image dataset (SH dataset) of Schistoscope-captured microscopic images of filter membranes prepared from spiked and clinical samples. The corresponding ground-truth images were created by manually annotating *S. haematobium* eggs in the captured images. Expert parasitologists carried out this task using the coco annotator tool [33]. For the creation of the ground-truth images, we applied the following principles:

1. Annotation of the exact boundary pixels of the *S. haematobium* eggs was not strictly enforced due to the limitation posed by the size of the eggs;
2. The pixel values of the background and artifacts in the ground-truth image were labelled as ‘0’ and the eggs as ‘1’;
3. There were few *S. mansoni* eggs found in the images of the clinical urine samples and their pixel values were labelled as ‘1’;
4. Pixels of partially cut eggs at the edges of the images were labelled as ‘1’;
5. The region of the eggs covered by artifacts was labelled as ‘0’.

A deep neural network (DNN) based on a UNET architecture [34] was trained for the segmentation of *S. haematobium* egg pixels using the SH dataset. The SH dataset was split into 70%, 15% and 15% to train, validate and test the automated system, respectively, and the deep neural network was trained for 16 epochs using Google Colaboratory’s Tesla P100-based servers. During the training stage, the image was resized to 512 × 512 pixels and the Adam solver was applied with a learning rate of $1\times 10^{-5}$ The momentum and the decay coefficient were set to 0.9 and $1\times 10^{-8}$, respectively. All the weights were initialized from a Gaussian distribution with a mean of 0 and a standard deviation of 0.02. The batch size was initialized to 8. After training, the test set was applied to the trained model and the segmentation performance was compared to the ground truth using the dice similarity coefficient [35] as metric. 

We developed a linear regression model for egg count estimation using the pixel area of each connected component and its corresponding actual egg count in the ground-truth image. The derived model was applied estimating the egg counts per image in the segmented mask images of the test set. We compared the results with the actual egg count per image using mean absolute error (MAE) and root mean squared error (RMSE) as metrics.

## 3. Results and Discussion

### 3.1. Sample Stage XY Position Repeatability

**Figure 3:**
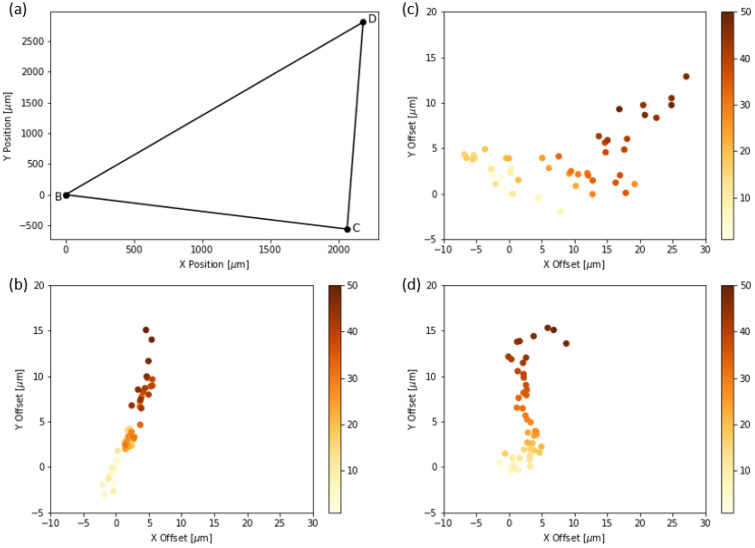
XY positioning accuracy. (**a**) the path taken by the sample stage. (**b**–**d**) the displacement of three eggs in the respective FoV from their initial positions in the captured frame from the first cycle.

We performed a sample stage XY position repeatability test [19,20] to quantitatively measure the positioning repeatability of the sample stage in the X- and Y-axis. We imaged *S. haematobium* eggs spread out across three adjacent FoVs and measured the accuracy with which we could repeatedly center the microscope objective over these different FoVs. We selected eggs located approximately 2000 µm to 3000 µm apart and programmed the auto-scanning system to repeatedly cycle between them 50 times and capture a single 1520 × 2028 pixels image upon arriving at each FoV. We then estimated the positioning error across the 50 cycles by calculating the number of pixels (and hence microns) by which subsequent frames are displaced from the first frame. A displacement of zero would indicate that the stage returned exactly to the starting position. The path taken by the sample stage is shown in Figure 3a, where the three vertices are the locations of the eggs in each FoV. Figure 3b–d show XY positioning errors for each egg. The color scale corresponds to the motion cycle number, indicating the order in which the data were acquired. The first data point is yellow and the last is brown. The colors are not distributed randomly, which indicates that there is a systematic drift. The estimated drift after 50 motion cycles of the three eggs from their initial positions were 11.17 µm, 13.68 µm and 11.75 µm, respectively, which is small relative to the size of the FoV. 

### 3.2. Imaging Performance

We evaluated the quantitative imaging performance of the Schistoscope by obtaining the resolution limit of the optical setup. Additionally, a qualitative comparison was performed between images taken by our device and images of the same FoV taken by a conventional microscope (BRESSER Science Infinity Microscope) equipped with a plan-achromatic objective (10× magnification and 0.25 numerical aperture). 

**Figure 4:**
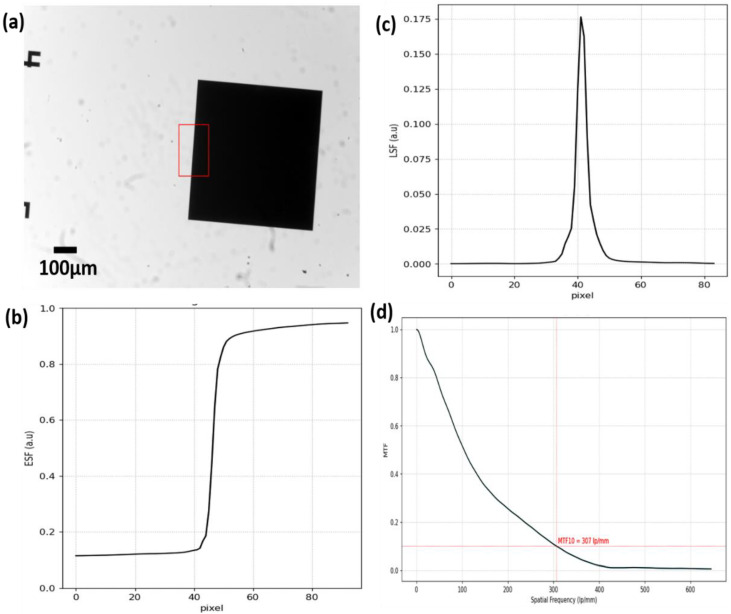
Resolution limit of the Schistoscope. (**a**) Slanted-edge image with selected rectangular region of interest (**b**) Edge spread function curve (**c**) Line spread function curve (**d**) Modulation transfer function curve with a resolution limit of 307 lp/mm.

We adopted the ISO 12233 slanted-edge technique [36], which provides a fast and efficient way of estimation and Modulation transfer function (MTF). First, we registered a slanted-edge image (derived from a standard USAF 1951 resolution target) using the Schistoscope. Next, we selected a rectangular region of interest (ROI) in the image with a step edge (Figure 4a). The device’s edge spread function (ESF) was then calculated by taking the response of the line perpendicular to the edge. Then we obtained the derivative of the ESF which is the line spread function (LSF). The MTF was derived by performing a one-dimensional Fourier transform of the LSF. The ESF, LSF and MTF curves are shown in Figure 4b–d. It was observed from the MTF curve that the limiting resolution (MTF10) of the device is 307 lp/mm (3.26 microns), which is in reasonable agreement with the Rayleigh theoretical value of 3.35 microns (assuming NA 0.1 and center wavelength 550 nm). Thus, the optical setup is more than sufficient to image the *Schistosoma* eggs with sizes within the bounds of 110 − 170 × 40 − 70 μm.

**Figure 5:**
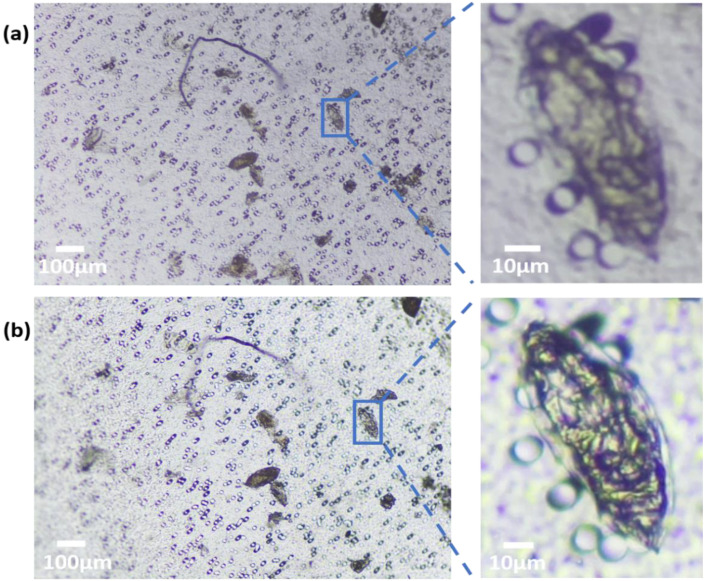
Optical performance of the Schistoscope. (**a**) Schistoscope (NA 0.1) and (**b**) conventional microscope (NA 0.25) images of fecal smear containing *Schistosoma haematobium* eggs. Enlarged ROIs show similar optical qualities.

**Figure 6:**
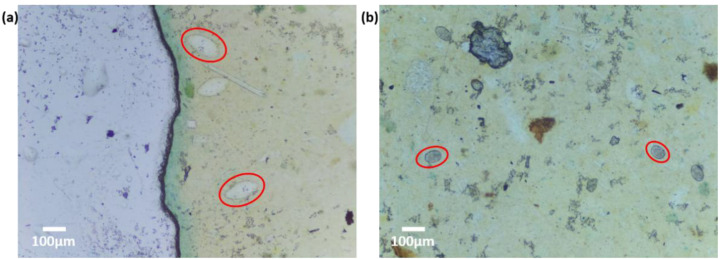
Captured images of intestinal parasites using the Schistoscope. Fecal smears of (**a**) region of interest showing *Schistosoma mansoni* eggs. (**b**) region of interest showing hookworm eggs.

In the qualitative comparison between the Schistoscope and a conventional microscope, we captured the same FoV on a sample slide containing *S. haematobium* eggs using both systems (Figure 5a,b). Despite the markedly superior optical characteristics of the conventional microscope, the overall FoV, field flatness, and visual perception of the two systems are not so different. A magnified region of interest is also presented for detailed comparison. Although the conventional microscope has an improved depth of focus and higher contrast, the quality of the Schistoscope image is acceptable as the terminal spine of the *S. haematobium* eggs and the lateral spine of the *S. mansoni* eggs (Figure 2) could be easily identified by a human reader. To further demonstrate the ability of the Schistoscope to aid in the diagnosis of intestinal parasites effectively, we used the device to image fecal smear containing eggs of *S. mansoni* and hookworm eggs. As can be clearly seen in Figure 6a,b, the Schistoscope device also can optically resolve the eggs of these intestinal parasites.

### 3.3. Performance Evaluation of S. haematobium Egg Detection Algorithm

**Figure 7:**
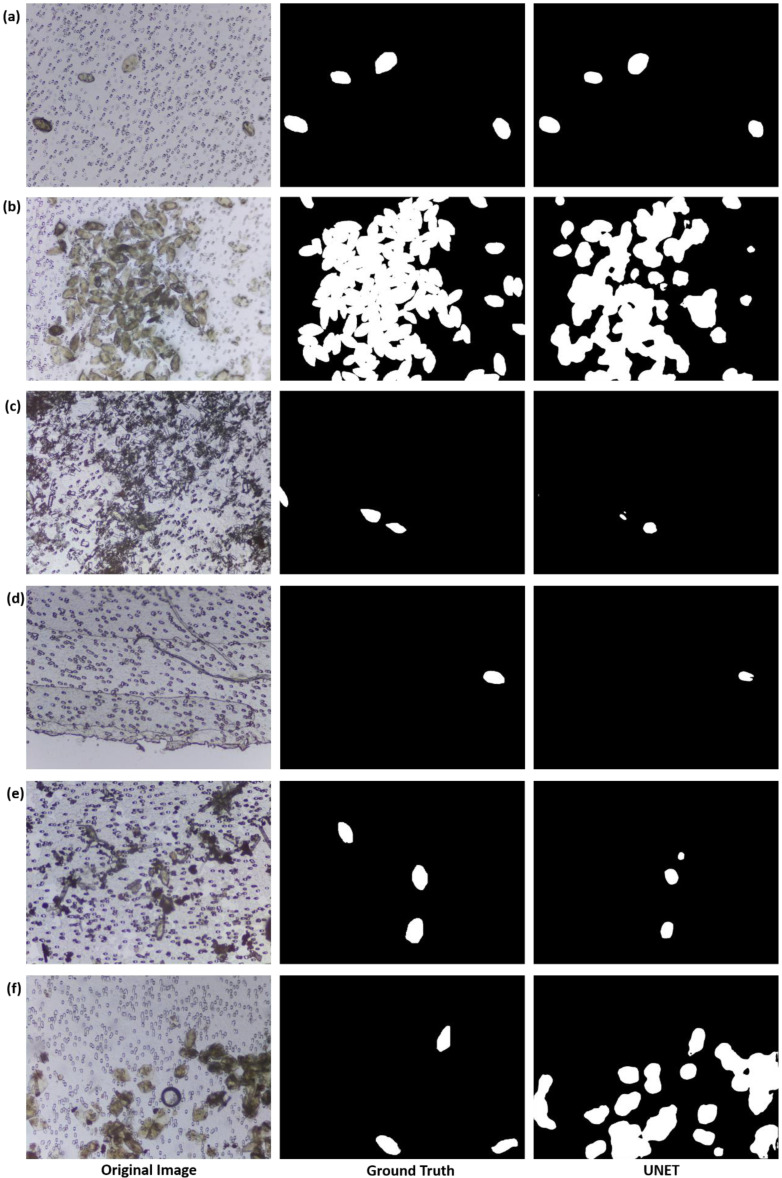
Visual comparison of semantic segmentation of images in test dataset (**a**,**b**) sample images from spiked urine samples (**c**–**f**) sample images from clinical urine samples.

We created an SH image dataset consisting of 5198 microscopic images of urine filter membranes (986 and 4212 images from spiked and clinical urine samples, respectively), along with their respective ground-truth images with 6437 annotated *S. haematobium* eggs (4776 and 1661 eggs in spiked and clinical urine samples images, respectively). Although images from the clinical samples had fewer or in some cases no eggs present compared to images of the spiked samples, they still contained artifacts such as crystals, glass debris, air bubbles, fabric fibers and human hair (selected images shown in Figure 7c,f), thus increasing the robustness of the dataset and the difficulty of the automatic egg detection task.

To address this challenge, we applied the trained UNET model for the semantic segmentation of *S. haematobium* eggs present in images of the test dataset. In the qualitative segmentation results of images in the test dataset (Figure 7), we observed that the deep-learning model performed better in the segmentation of eggs in images from the spiked urine samples (Figure 7a,b) than in clinical samples (Figure 7c–f). Probable reasons for this difference may be that a higher percentage of eggs in the SH dataset were from the spiked samples, and the high presence of artifacts in the images captured from the clinical samples could have caused segmentation errors. An example of such an error can be seen in Figure 7f, where many uric acid crystals are present in the image. The similarity between morphological features of the crystals and the *S. haematobium* eggs causes the deep-learning model to falsely identify the crystals as eggs.

**Figure 8:**
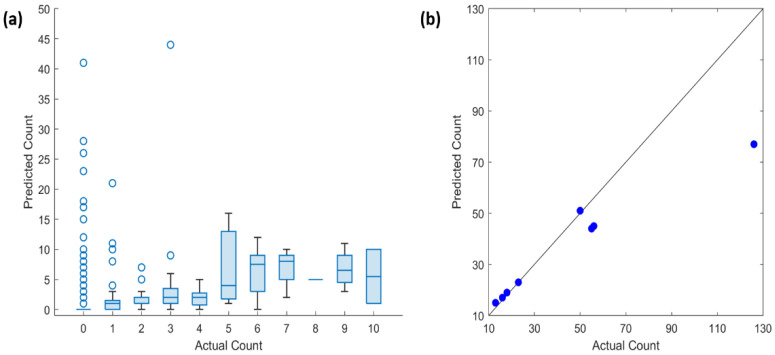
Quantitative result of the predicted egg counts per captured FoV image (**a**) visual summary of the egg counts in test images with 0–10 actual egg count (~98% of the test images) (**b**) Scatter plot of test images with actual egg counts greater than 10.

In the quantitative results, we obtained a dice similarity coefficient of 0.44. The observed low dice similarity coefficient in the test data could be due to the following assumptions: (i) the non-strict-enforcement of exact boundary conditions in the annotation of the *S. haematobium* eggs in the ground-truth images; and (ii) poor segmentation performance of the UNET in the difficult clinical images with egg-like artifacts (uric acid crystals). We also estimated the egg count per captured FoV image using a linear prediction model with the area of the segmented egg pixels as the independent variable. We obtained a MAE and RMSE of 1.21 and 4.08, respectively. A box plot shows a visual summary of the estimated egg counts in test images with 0–10 actual egg count (~98% of the test images) (Figure 8a). An increased number of outliers above the maximum whisker of the box plot is observed in the set of images with 0 or 1 actual egg count, which are predominantly images with artifacts from clinical urine samples. From this result, we infer that the automated detection model could satisfy to the 80% sensitivity diagnostic requirement specified in the WHO Target Product requirement for the diagnosis of schistosomiasis [37]. 

Furthermore, there is a small difference between the average predicted egg count in each box and actual egg count, which is evident in the MAE value of the test dataset. Figure 8b shows the scatter plot of test images with actual egg count greater than 10. It is observed that the deviation from the line of perfect agreement (black line) increases with higher number of actual egg count per image. This is a result of the increasing occurrence of overlapping eggs with an increasing number of actual egg count per image. Thus, we believe a logarithmic model or an egg counting algorithm that explores the eggs’ morphological properties (e.g., egg size and shape) might be a suitable solution to this problem.

## 4. Conclusions

We developed an optical diagnostic device called the Schistoscope, which incorporates an automated Z-axis movement for autofocusing a sample based on a Laplacian sharpness metric, as well as an automated XY movement of the sample stage for automated grid scanning. Our experiments showed that the optical system has comparable performance with conventional microscopes. We demonstrated the automatic detection of *S. haematobium* eggs by creating a robust *S haematobium* egg image dataset containing over 5000 FoV images of filtered spiked and clinical urine samples from field settings. We trained a deep neural network model for the semantic segmentation of the *S. haematobium* eggs prior to egg count estimation using a linear model based on the area of the segmented pixels. Although urine artifacts present in the images from the clinical sample posed a challenge, the algorithm clearly identified the eggs in the image, demonstrating that the quality of the images is suitable for automatic detection of *Schistosoma* eggs in line with the current diagnostic reference standard. High-quality microscopy images of *S. haematobium*, *S. mansoni* and hookworm eggs were captured using the device, and the eggs were clearly identified in captured digital images by microscopists.

Therefore, it is evident that combining automated image acquisition with a suitable artificial intelligence algorithm in the device for diagnosis will significantly increase its potential as a diagnostic tool in resource-limited settings. Manuscripts describing the outcome of a population-based survey, and validating the diagnostic performance of the Schistoscope for the detection of *S. haematobium* eggs in urine samples in a low-resource field setting, are currently in progress.

In conclusion, the Schistoscope was presented to the national technical working committee on the eradication of schistosomiasis in Nigeria. Possible potential benefits of the Schistoscope discussed include point-of-need diagnosis and drug efficacy monitoring, which could mitigate waste of human, material and financial resources. Ongoing discussions with local private and public partners aim to explore ways to integrate the Schistoscope into active schistosomiasis elimination and control programs in Nigeria.

## Data Availability

*Schistosoma haematobium* image dataset is available from the Zenodo Repository 10.5281/zenodo.6467268.
